# Alcohol promotes mammary tumor growth through activation of VEGF-dependent tumor angiogenesis

**DOI:** 10.3892/ol.2014.2146

**Published:** 2014-05-16

**Authors:** YANMIN LU, FANG NI, MEI XU, JINLIAN YANG, JI CHEN, ZHUO CHEN, XINYI WANG, JIA LUO, SIYING WANG

**Affiliations:** 1 Department of Pathophysiology, Anhui Medical University, Hefei, Anhui 230032, P.R. China; 2 Department of Hepatobiliary Surgery, Clinical Nutrition Center, Clinical Nutrition and Metabolism Key Laboratory, Binzhou Medical University Hospital, Binzhou, Shandong 256603, P.R. China; 3 Department of Internal Medicine, College of Medicine, University of Kentucky, Lexington, KY 40536, USA

**Keywords:** alcohol, breast cancer, angiogenesis, endothelial cells

## Abstract

Alcohol consumption has been recognized as a risk factor for breast cancer. Experimental studies demonstrate that alcohol exposure promotes the progression of existing mammary tumors. However, the mechanisms underlying this effect remain unclear. In the present study, the role of vascular endothelial growth factor (VEGF) in alcohol promotion of breast cancer development was investigated using a mouse xenograft model of mammary tumors and a three-dimensional (3D) tumor/endothelial cell co-culture system. For the mouse xenograft model, mouse E0771 breast cancer cells were implanted into the mammary fat pad of C57BL6 mice. These mice were exposed to alcohol in their drinking water. For the 3D co-culture system, E0771 cells and MDA-MB231 breast cancer cells were co-cultured with SVEC4-10EE2 and human umbilical vein endothelial cells, respectively. The results demonstrated that alcohol increased tumor angiogenesis and accelerated tumor growth. Furthermore, it appeared that alcohol induced VEGF expression in breast cancer cells *in vitro* and *in vivo*. Blocking VEGF signaling by SU5416 inhibited tumor angiogenesis in the 3D tumor/endothelial cell co-culture system. Furthermore, injection of SU5416 into mice inhibited alcohol-promoted mammary tumor growth *in vivo*. These results indicate that alcohol may promote mammary tumor growth by stimulating VEGF-dependent angiogenesis.

## Introduction

Breast cancer is the most common malignant tumor in females worldwide and is the second leading cause of cancer-related mortality in the USA (1,2). There are multiple risk factors for breast cancer, including genetic, reproductive/hormonal, environmental and other lifestyle factors (3). Epidemiological studies indicate that alcohol consumption increases the risk of breast cancer in a dose-dependent manner (4–9). In addition, alcohol enhances the growth of existing breast tumors and promotes metastasis (10–12). However, the mechanisms underlying these effects remain unclear.

Tumor growth and metastasis are dependent on angiogenesis. Vascular endothelial growth factor (VEGF) is one of the most important known factors which stimulates vasculogenesis and angiogenesis. VEGF has an important role in tumor angiogenesis via promoting proliferation, migration, stabilization and survival of endothelial cells as well as tumor cells (13,14). It has been demonstrated that the VEGF expression in breast cancer tissues is significantly higher than that in the adjacent normal tissues (15). We previously demonstrated that alcohol promoted angiogenesis and induced the expression of monocyte chemotactic protein-1 (MCP-1). However, MCP-1 only partially mediated the effect of alcohol, that is, blocking MCP-1 signaling only partially reversed the effect of alcohol on angiogenesis and mammary tumor growth (16). Alcohol-induced tumor promotion may be mediated by multiple factors and signaling pathways. Considering the important role of VEGF in tumor angiogenesis and progression, we hypothesized that VEGF signaling is involved in alcohol promotion of tumor angiogenesis and mammary tumor growth. In the present study, we utilized *in vitro* and *in vivo* model systems to address this hypothesis.

## Materials and methods

### Materials

Ethanol, fibrinogen, aprotinin and thrombin were purchased from Sigma-Aldrich (St. Louis, MO, USA), and SU5416 was purchased from Calbiochem (San Diego, CA, USA). Rat anti-mouse CD31 monoclonal antibody was obtained from BD Biosciences (San Diego, CA, US), while anti-VEGF antibody was obtained from Santa Cruz Biotechnology, Inc. (Dallas, TX, USA). Cytodex 3 beads were obtained from Amersham Pharmacia Biotech Inc. (Piscataway, NJ, USA).

### Cell culture

Mouse mammary adenocarcinoma cell line (E0771) was provided by Dr. Enrico Mihich (Roswell Park Cancer Institute, Buffalo, NY, USA) and maintained in DMEM (Gibco-BRL, Carlsbad, CA, USA) supplemented with 10% fetal bovine serum (FBS; Hyclone, Logan, UT, USA), penicillin (100 U/ml;), streptomycin (100 U/ml) and amphotericin B (0.25 mg/ml) at 37°C with 5% CO_2_ (GBCBIO™ Technologies, Guangzhou, China). MDA-MB231 breast cancer cells and SVEC4-10EE2 murine endothelial cells (both American Type Culture Collection, Manassas, VA, USA) were grown in DMEM medium containing 10% FBS and 100 U/ml penicillin and streptomycin at 37°C with 5% CO_2_. Human umbilical vein endothelial cells (HUVECs; American Type Culture Collection) were isolated from fresh human placentas with type I collagenase (1 mg/ml) and grown in Clonetics Endothelial Cell Growth Medium-2 (EGM-2; Lonza, Walkersville, MD, USA). HUVECs were used between passages 3 and 10.

### Animals and alcohol exposure

Female C57BL/6 mice (5–6 weeks old) were purchased from the Experimental Animal Center of Anhui Province (Hefei, China). All procedures were conducted according to the Guidelines of the Animal Welfare Act approved by the Institutional Animal Care and Use Committee of the Anhui Medical University (Hefei, China). The mice were placed on a standard chow diet and were allowed to acclimatize for one week prior to starting the study. The paradigm of alcohol exposure has been previously described (16). Briefly, mice were divided into two groups and fed with standard chow *ad libitum*. In the control group, the mice were provided with regular drinking water (n=20). In the alcohol-exposed group (n=18), the mice received repeated cycles of chronic intermittent ethanol (2% v/v) exposure (12 h/day, 8:00pm–8:00am) for three weeks. The consumption of regular water or alcohol-containing water in the two groups was monitored daily and no significant difference in the liquid intake between the control and ethanol group was identified. The average consumption of water or ethanol-containing water for each mouse was ~4 ml/day. The study was approved by the ethics committee of Anhui Medical University.

### Mouse tumor xenograft model

E0771 mouse breast cancer cells are syngeneic to C57BL/6 mice. E0771 cells, implanted subcutaneously in C57BL/6 mice, are immunosuppressive and highly aggressive, invading locally into dermal layers and the peritoneum as well as distantly to the lung, and have characteristics which reflect the human disease (16,17). These cells have been extensively used for mouse tumor xenograft models. Briefly, three days following alcohol exposure, E0771 cells [2.5×10^5^ in 100 μl phosphate-buffered saline (PBS)] were injected into the secondary mammary fat pad of mice using a 23-gauge needle. Following implantation, the mice were continually provided normal drinking water or water containing 2% ethanol. The tumor size was monitored every three days. Two perpendicular dimensions of tumors were measured using a dial caliper and the tumor volume was calculated based on the formula: V=0.24a × b^2^ (a, the longest dimension and b, the shortest dimension). Following implantation, 24 days later the mice were sacrificed and the tumors were harvested for further detection (16).

To investigate the role of VEGF in alcohol-induced tumor promotion, an inhibitor of the VEGF receptor, Z-3-[(2, 4-dimethylpyrrol-5-yl) methylidenyl]-2-indolinone (SU5416; Sigma-Aldrich) was injected into the mice. One day following alcohol exposure, animals received intraperitoneal injection of SU5416 [10 mg/kg in 100 μl, Su5416 was dissolved in ethanol and diluted in 5% Tween-80 (Amresco LLC, Solon, OH, USA) and 5% PEG-400 (Shandong Lunan Chemical Technology Co., Ltd, Tengzhou City, China) prior to injection] every three days. The dosage has been previously demonstrated to effectively inhibit VEGF signaling in mice (18). The tumor size was monitored every three days.

### Immunohistochemistry (IHC) and evaluation of average microvessel density (AMVD)

The procedure for immunohistochemical analysis was performed as described previously (19). Tumor tissues were sectioned at the thickness of 4 μm. The sections were incubated in methanol (0.3% H_2_O_2_) for 30 min and treated with 0.1% Triton X-100 (Amresco LLC) for 10 min. The sections were washed with PBS three times and blocked with 1% bovine serum albumin (BSA; Amresco LLC) and 0.01% Triton X-100 for 1 h at room temperature. The sections were incubated with anti-VEGF antibody (1:100) or anti-CD31 (1:50) antibody overnight at 4°C. Negative controls were performed by staining with isotype-matched IgG or PBS. Following rinsing in PBS, the sections were incubated with goat anti-rat biotinylated monoclonal secondary antibodies (Vector Laboratories Inc., Burlingame, CA, USA) for 1 h at room temperature. The sections were washed three times with PBS, then incubated in avidin-biotin-peroxidase complex (1:100 in PBS; Vector Laboratories Inc., Burlingame, CA, USA) for 1 h and developed in 0.05% 3,3′-diaminobenzidine (Sigma-Aldrich) containing 0.003% H_2_O_2_ in PBS. The tumor microvessels that were visualized by CD31 IHC were examined under a microscope (Olympus CX31; Olympus Corporation, Tokyo, Japan). Ten random fields were quantified and the AMVD was expressed as the number of microvessels/mm^2^ area.

### Three-dimensional (3D) endothelial tumor cell co-culture system

To investigate the effect of alcohol on tumor angiogenesis, a 3D tumor/endothelial cell co-culture was performed as described previously (16,20). In this model, endothelial cells were cultured on a fibrin gel bead system alone or with tumor cells to form a 3D capillary tube-like network. Briefly, HUVECs or SVEC4 cells were trypsinized and the cells (1×10^6^) were mixed with cytodex beads (3×10^3^) in a 4 ml medium (EGM-2 for HUVECs and DMEM for SVECs). The mixtures were incubated at 5% CO_2_ at 37°C and gently shaken every 20 min for 4 h. Following this, 4 ml of fresh medium was added to the tubes and the incubation continued for another 4 h. The mixtures of cells/cytodex beads were transferred to 25 ml tissue culture flasks and incubated overnight to allow the bead-non-attached cells to adhere to the flask. Following incubation, the mixtures of cells/cytodex beads were transferred to a 50 ml centrifuge tube and washed three times with 20 ml of Ca^2+^- and Mg^2+^-free PBS. Then, the beads with adherent cells were resuspended in medium [2.5 mg/ml fibrinogen, 0.15 U/ml aprotinin (Amresco LLC), pH 7.4]. Following this, 0.5 ml of the fibrinogen/bead suspension was added to 24-well cell culture plates which were pre-coated with 0.625 U of thrombin (Sigma-Aldrich). The fibrinogen/bead solution was allowed to coagulate for 5 min at room temperature and was then cultured in 5% CO_2_ at 37°C for 20 min. The resulting fibrin gels contained endothelial cells (HUVECs or SVECs) adhering to the beads. Then, 1 ml of medium (0.15 U/ml aprotinin) was added to each well to equilibrate with the fibrin clot for 30 min at 37°C and 5% CO_2_. The medium was removed and replaced with 1 ml of fresh medium (0.15 U/ml aprotinin). For co-culture of endothelial cells/breast tumor cells, E0771 or MDA-MB231 breast cancer cells (2×10^4^ or 4×10^4^, respectively) were layered on top of the fibrin gels. The medium was changed every day.

### In vitro alcohol exposure

A method utilizing sealed containers was used to maintain alcohol concentrations in the cell culture system (21). Briefly, the appropriate amount of ethanol (using 95% ethanol stock) was added to the culture medium to reach the desired concentration (0.2%). The cell culture plates were placed in a sealed plastic container. In each container, a water bath with 200 ml 0.2% ethanol was deposited in order to maintain the ethanol concentration. Prior to sealing each container, CO_2_ (60 ml) was injected. The containers were placed in a humidified environment and maintained at 37°C with 5% CO_2_. With this method, ethanol concentrations were maintained constantly over time in a cell culture medium (21).

### Immunoblotting

The immunoblotting was performed as previously described (22). Briefly, aliquots of the protein samples (30 μg) were separated on an SDS-polyacrylamide gel (Sigma-Aldrich) and were transferred to nitrocellulose membranes. The membranes were blocked with 5% BSA solution [pH 7.4, 0.05% Tween-20 (Amresco LLC) in PBS] for 1 h at room temperature. Anti-VEGF antibody (1:500) was added to the membranes (Millipore, Billerica, MA, USA) for 1 h at room temperature. Membranes were probed with goat anti-rat monoclonal horseradish peroxidase-conjugated secondary antibody (Amersham Life Science, Arlington Heights, IL, USA). The signals were detected by using the enhanced chemiluminescence method (Amersham Life Sciences) and were exposed to X-ray film for autoradiography. The membranes were stripped with a stripping buffer for 15 min at room temperature and immunoblotted with a rabbit anti-mouse actin monoclonal antibody (Santa Cruz Biotechnology, Inc.). The images were scanned and the signal intensity was quantified with Image J software (NIH, Bethesda, MD, USA).

### VEGF enzyme-linked immunosorbent assay (ELISA)

ELISA was performed in 100-μl volumes in triplicate using commercial kits for VEGF, according to the manufacturer’s instructions (R&D Systems, Minneapolis, MN, USA). The plates were read at 450 nm on an ELx800 absorbance microplate reader (Bio-Tek Instruments Inc., Winooski, VT, USA).

### Statistical analysis

The data were analyzed using SPSS 17.0 software (SPSS, Inc., Chicago, IL, USA). All data are represented as the mean ± SEM. Differences among the treatment groups were examined using analysis of variance. In the cases where significant differences were detected, specific post-hoc comparisons between the treatment groups were examined with Student-Newman-Keuls tests. In a number of the experiments, the results were analyzed by an unpaired Student’s t-test. P<0.05 was considered to indicate a statistically significant difference.

## Results

### Ethanol induces VEGF expression and tumor angiogenesis

**Figure 1:**
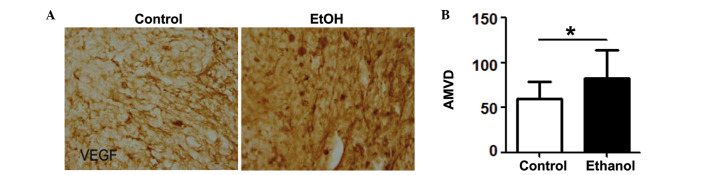
Effect of alcohol exposure on VEGF expression and angiogenesis in mice. (A) E0771 mouse breast cancer cells were implanted in mammary fat pads of C57BL6 mice. The mice were exposed to alcohol in drinking water for two weeks. Mice were sacrificed and mammary tumors were dissected and sectioned for VEGF IHC as described in Materials and methods. (B) Tumor microvessels were identified by CD31 IHC and the AMVD was quantified and expressed as the number of microvessels/mm^2^ area. The results are represented as the mean ± SEM of 20–22 animals. ^*^Denotes a statistically significant difference (P<0.05). VEGF, vascular endothelial growth factor; IHC, immunohistochemistry; AMVD, average microvessel density.

**Figure 2:**
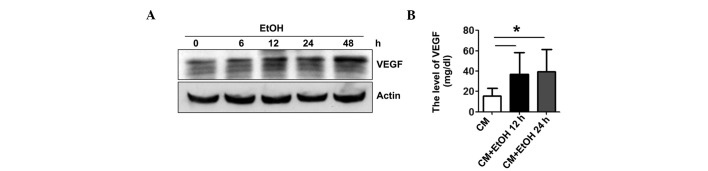
Effect of alcohol on VEGF expression in E0771 cells. (A) E0771 cells were exposed to alcohol (0 or 0.2%) for the indicated times and cell lysates were collected. The expression of VEGF was determined by western blotting as described in Materials and methods. The experiment was replicated three times. (B) E0771 cells were exposed to alcohol (0 or 0.2%) for 12 or 24 h. The CM was collected for the analysis of VEGF by enzyme-linked immunosorbent assay. The experiment was replicated three times. ^*^Denotes a statistically significant difference (P<0.05). VEGF, vascular endothelial growth factor; CM, conditioned medium; EtOH, ethanol.

The effect of alcohol on VEGF expression and tumor angiogenesis was investigated in a mouse xenograft model of mammary tumors. As demonstrated in Fig. 1A, the VEGF immunoreactivity in the mammary tumor tissues of mice exposed to alcohol was higher than that of the control mice. The AMVD was also examined using CD31 (a marker of endothelial cells) IHC. The quantification data are presented in Fig. 1B. Alcohol consumption significantly increased the AMVD in mammary tissues. To confirm alcohol stimulation of VEGF expression, we examined the effect of alcohol on E0771 mouse breast cancer cells in culture. Alcohol exposure upregulated VEGF expression in E0771 cells (Fig. 2A) and increased the secretion of VEGF to the culture medium (Fig. 2B).

### Ethanol promotes tumor angiogenesis

**Figure 3:**
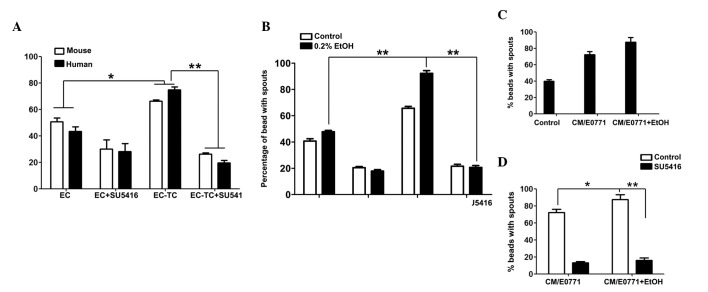
Effect of alcohol on tumor angiogenesis *in vitro*. (A) The 3D tumor/endothelial cell co-culture system was set up as described in Materials and methods. Endothelial cells (SVECs or HUVECs) attached to cytodex beads were suspended in the fibrin gel containing SU5416 (0 or 2 μM) and breast cancer cells (E0771 or MDA-MB231) were placed on top of the gel. The endothelial cell sprouting was examined and recoded under a microscope following 12 h in culture. The percentage of beads with endothelial sprouts was calculated. EC, SVECs or HUVECs; TC, E0771 or MDA-MB231 cells. (B) SVEC/E0771 cell co-culture with/without SU5416 (2 μM) was exposed to alcohol (0 or 0.2%) for 12 h. Following this, the percentage of beads with endothelial sprouts was quantified. The experiment was replicated three times. (C) E0771 cells were maintained in a medium containing 1% FBS and exposed to alcohol (0 or 0.2%) for 24 h. The CM was collected and SVECs were incubated with this CM for 12 h. Then, the percentage of beads with endothelial sprouts was calculated. (D) The CM was collected from alcohol (0 or 0.2%)-treated E0771 cells and SU5416 (0 or 2 μM) was added to the medium. SVECs were then incubated with this conditioned medium for 12 h. The experiments were replicated three times. Each data point represents the mean ± SEM of three replicates.^*^P<0.05 and ^**^P<0.01. 3D, 3-dimensional; EC, endothelial cells; TC, tumor cells; CM, conditioned medium; EtOH, ethanol.

We hypothesized that alcohol increased VEGF production in breast cancer cells and stimulated angiogenesis of endothelial cells. A previously described 3D angiogenic model was utilized to test this hypothesis. With this system, we previously demonstrated that breast cancer cells (E0771 cells or MDA-MB231 cells) significantly increased the vascular sprout formation of endothelial cells (SVECs or HUVECs) (16). In the present study, VEGF signaling in tumor/endothelial cell co-culture was blocked using SU5416, an inhibitor of VEGF receptor 2 (VEGFR2). As demonstrated in Fig. 3A, inclusion of breast cancer cells (E0771 or MDA-MB231) in this 3D co-culture system increased endothelial cell sprouting, which was indicative of enhanced angiogenesis. Treatment with SU5416 completely blocked the promotion of angiogenesis mediated by breast cancer cells. Alcohol exposure did not increase endothelial cell sprouting when the endothelial cells were cultured alone; however, it significantly increased endothelial cell sprouting in SVEC/E0771 co-culture (Fig. 3B). More importantly, SU5416 blocked alcohol-stimulated angiogenesis (Fig. 3B). To confirm the role of VEGF in alcohol-stimulated angiogenesis, the effect of conditioned medium collected from alcohol-treated E0771 cells on angiogenesis was examined. As demonstrated in Fig. 3C, E0771 cell-conditioned medium significantly increased endothelial cell sprouting and alcohol exposure further enhanced this effect. SU5416 completely blocked alcohol-stimulated angiogenesis. These results suggested that alcohol-enhanced tumor angiogenesis was mediated by VEGF signaling.

### SU5416 inhibits ethanol-promoted tumor growth in mouse tumor xenograft model

**Figure 4:**
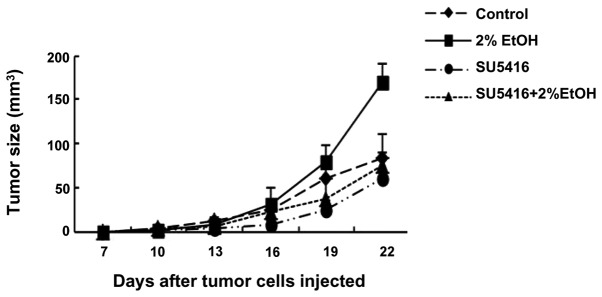
Effect of alcohol on mammary tumor growth *in vivo*. The implantation of E0771 cells in C57Bl6 mice and alcohol exposure was performed as described in Materials and methods. One day following E0771 cell implantation, mice received an intraperitoneal injection of SU5416 (0 or 10 mg/kg) every three days. The size of the mammary tumor was measured every three days as described in Materials and methods (n=12 for each treatment group). EtOH, ethanol.

A well-established mouse xenograft model of mammary tumors was utilized to validate the role of VEGF in alcohol-induced tumor promotion *in vivo*. As demonstrated in Fig. 4, alcohol consumption significantly promoted the growth of mammary tumors in C57BL6 mice. Injection of SU5416 inhibited alcohol-stimulated tumor growth. These results suggested that alcohol promotion of tumor growth may be mediated by VEGF-dependent angiogenesis.

## Discussion

Alcohol consumption promotes the growth and metastasis of breast cancer (10–12). However, the mechanisms underlying this effect remain unclear. VEGF has been implicated in tumor angiogenesis and progression. In the present study, it was demonstrated that alcohol increases tumor angiogenesis and accelerates tumor growth. Alcohol upregulates VEGF expression in breast cancer cells *in vitro* and *in vivo*. Blocking VEGF signaling inhibits alcohol-stimulated tumor angiogenesis in a 3D tumor/endothelial cell co-culture system. Furthermore, blocking VEGF signaling inhibited alcohol-accelerated mammary tumor growth in mice. These results suggest that VEGF-dependent angiogenesis has an important role in alcohol-mediated tumor promotion.

Consistent with epidemiological evidence, alcohol exposure promotes tumor progression and malignancy in animals (16,23,24,25). In the present study, it was demonstrated that alcohol at a moderate concentration (2% in drinking water during the dark cycle) enhances mammary tumor growth in C57BL6 mice. Our previous results and studies by others suggested that alcohol exposure may enhance angiogenesis (16,23,26,27). The mechanisms underlying alcohol-stimulated angiogenesis, however, are complex and unclear. Alcohol may directly target endothelial cells, or regulate the interaction between endothelial and tumor cells. To the best of our knowledge, our previous study was the first to demonstrate that alcohol promotes tumor/endothelial cell interaction and enhances tumor angiogenesis in a 3D co-culture of tumor/endothelial cells (16). In this earlier study, we demonstrated that alcohol stimulated MCP-1 secretion from mammary tumor cells, which enhanced endothelial angiogenesis. However, blocking MCP-1 signaling only partially reversed the effect of alcohol on tumor angiogenesis. Therefore, we hypothesized that other factors are responsible for mediating alcohol-stimulated angiogenesis.

VEGF is one of the most potent effectors of physiological and pathological angiogenesis, which has been proved to be an important factor for tumor occurrence, progression and metastasis of breast cancer (13). In the tumor microenvironment, VEGF is derived from various sources, including tumor cells, inflammatory and stromal cells, platelets and vascular cells (28). VEGF regulates multiple aspects of tumor angiogenesis through two high-affinity receptor tyrosine kinases, VEGFR1 (Flt-1) and VEGFR2/KDR (Flk-1), on endothelial cells. The binding of VEGF and VEGFR results in endothelial cell proliferation, migration, differentiation, tube formation and upregulation of vascular permeability (29). Furthermore, VEGF has been reported to be an indirect leukocyte migrating factor through inducing the expression of MCP-1 and IL-8 (30,31). In the present study, it was demonstrated that SU5416, a tyrosine kinase inhibitor of VEGFR2, not only blocks tumor cell-stimulated angiogenesis but also eliminates alcohol-mediated promotion of tumor angiogenesis (Fig. 3). Furthermore, SU5416 significantly inhibits alcohol-stimulated mammary tumor growth in mice. Therefore, this study confirms that VEGF has an important role in alcohol promotion of mammary tumor progression. Furthermore, it appears that the role of VEGF in alcohol-mediated tumor promotion is not tumor type-specific. It has been reported that moderate alcohol consumption increases the expression of VEGF and angiogenesis in a mouse xenograft model of melanoma (16). Multiple factors and signaling pathways may be responsible for alcohol promotion of mammary tumor progression and VEGF signaling appears to be an important one. Future studies are required to elucidate the underlying mechanisms whereby alcohol regulates the expression of VEGF.
